# A Tight Spot: How Personality Moderates the Impact of Social Norms on Sojourner Adaptation

**DOI:** 10.1177/0956797618815488

**Published:** 2019-01-23

**Authors:** Nicolas Geeraert, Ren Li, Colleen Ward, Michele Gelfand, Kali A. Demes

**Affiliations:** 1 Department of Psychology, University of Essex; 2 Department of Psychology, University of Maryland; 3 Centre for Applied Cross-Cultural Research, Victoria University of Wellington

**Keywords:** tightness–looseness, cultural adaptation, personality, social norms, sojourners

## Abstract

How do you navigate the norms of your new culture when living abroad? Taking an interactionist perspective, we examined how contextual factors and personality traits jointly affect sojourners’ adaptation to the host-country culture. We hypothesized that tightness (strong, rigidly imposed norms) of the host culture would be associated with lower levels of adaptation and that tightness of the home culture would be associated with higher levels of adaptation. Further, we proposed that the impact of tightness should be dependent on personality traits associated with navigating social norms (agreeableness, conscientiousness, and honesty-humility). We analyzed longitudinal data from intercultural exchange students (*N* = 889) traveling from and to 23 different countries. Multilevel modeling showed that sojourners living in a tighter culture had poorer adaptation than those in a looser culture. In contrast, sojourners originating from a tighter culture showed better adaptation. The negative effect of cultural tightness was moderated by agreeableness and honesty-humility but not conscientiousness.

In the 21st century, an era of globalization, people are crossing borders at an unprecedented rate. Around 250 million people are currently residing outside their country of birth (United Nations, Department of Economic and Social Affairs, Population Division, 2015), and more than 55 million are sojourners or short-term travelers, such as expatriates and international students (Finaccord, 2014; ICEF Monitor, 2015). These global trends point to an urgent need to understand the dynamics of intercultural contact and the factors that facilitate or impede success and adaptation.

Multicultural experiences can bring benefits to individuals, groups, and organizations, enhancing self-esteem, self-efficacy, and creativity (Geeraert & Demoulin, 2013; Leung, Maddux, Galinsky, & Chiu, 2008; Milstein, 2005); improving intergroup relations (Tadmor, Hong, Chao, Wiruchnipawan, & Wang, 2012); adding productive capacity; and increasing innovative ideas (Maddux & Galinsky, 2009; Tadmor, Satterstrom, Jang, & Polzer, 2012). At the same time, crossing cultures brings serious risks: psychological distress (Kawai & Strange, 2014; Szabo, Ward, & Jose, 2016); impaired performance (Rienties & Tempelaar, 2013); early return, defined as prematurely returning home (Demes & Geeraert, 2015); hostile intercultural encounters (Mahajan, 2011; Ward, Masgoret, & Gezentsvey, 2009); and financial, social, and reputational challenges for organizations (Harrison & Shaffer, 2005; McNulty & Tharenou, 2004). Successful sojourning, defined as adapting psychologically (feeling well) and socioculturally (doing well), maximizes the benefits and minimizes the risks of multicultural experiences. In short, sojourner success depends on adaptive responses that maintain well-being, ensure the acquisition of appropriate cultural skills, and achieve the educational or occupational objectives of the sojourn.

Despite the critical importance of understanding sojourner adaptation, psychological research has been limited in its scope. Whereas there is an abundance of empirical evidence on the intrapersonal factors (including personality) and interpersonal factors that predict sojourner adaptation (Bierwiaczonek & Waldzus, 2016; Wilson, Ward, & Fischer, 2013), the influence of contextual factors on adaptive outcomes has received only limited attention (Ward & Geeraert, 2016). Indeed, few cross-cultural studies have examined cultural-level and sociopolitical factors (Berry, Phinney, Sam, & Vedder, 2006; Geeraert & Demoulin, 2013; Kashima & Abu-Rayya, 2014); however, the relationship between social norms and sojourner adaptation has not yet been investigated. Moreover, research that considers the interaction effects of individual and contextual factors on adaptive outcomes is virtually nonexistent. Drawing on the success of the person–situation interactionist perspective in explaining many other phenomena in psychological science, such as creativity (Hirst, Van Knippenberg, & Zhou, 2009), leadership effectiveness (Ng, Ang, & Chan, 2008), and well-being (Conger, Conger, & Martin, 2010), we designed this study to fill a major void in sojourner research by advancing the first interactionist study to examine the relationship between social norms and cultural adaptation and how personality moderates this relationship.

Specifically, we propose that the culture of the destination, the culture of origin, and sojourner personality interact in important ways to predict cultural adjustment. With respect to the destination, we advance a new theory on how the normative context of countries, specifically the strength of social norms (tightness), affects adaptation outcomes. Tight cultures—that is, cultures that have strong norms and little tolerance for deviance—restrict the range of behaviors that are appropriate in everyday situations (Gelfand et al., 2011; Harrington & Gelfand, 2014). Sojourners moving to tight cultures will invariably have stronger norms to adhere to, making their adjustment more difficult than for sojourners moving to loose cultures. Moreover, when sojourners move to other cultures, it is inevitable that they will not understand all of the norms that they must comply with, and thus they will inadvertently violate them. Individuals from tight cultures tend to have much more negative attitudes toward norm-violating individuals who disrupt the social order and who thus will be either directly reprimanded or indirectly punished (e.g., through ostracism). By contrast, loose cultures, with weaker norms and a higher tolerance for deviance, allow a much wider range of behaviors and are more open toward norm breakers. Our first aim of this research, therefore, was to examine, for the first time, the hypothesis that sojourners who venture to a tighter culture will have poorer adaptation than those who go to a looser culture (Hypothesis 1).

It may also be the case that the normative context of the culture of origin plays an important role in cultural adaptation. Compared with those who have been socialized in loose cultures, individuals who have been socialized in tight cultures are likely to have a stronger *normative radar*—they have higher self-monitoring and can more easily detect the strength and importance of social norms in their environment (Gelfand et al., 2011). Moreover, they have greater self-control and better self-regulation in response to normative requirements (Gelfand et al., 2011; Mu, Han, & Gelfand, 2017), which have been shown to be related to both psychological and sociocultural dimensions of cultural adaptation (Kosic, Mannetti, & Sam, 2006; Wong, Lam, Yan, & Hung, 2004); thus, we hypothesized a positive relationship between home-culture tightness and cultural adaptation (Hypothesis 2).

We also examined whether the negative effect of host-cultural tightness on adaptation is moderated by the strength of norms of the sojourners’ original culture. To the extent that people from cultures with strong norms are better able to adapt to other cultures, compared with people from loose cultures, we hypothesized that home-culture tightness buffers the negative effect of host-culture tightness on sojourner adaptation (Hypothesis 3). Notably, past research has lacked the appropriate design to examine these important questions. Researchers typically have relied on *unilateral samples*—for example, sojourners who originated from or traveled to a single country, making an examination of the country-level context difficult. In the current research, we analyzed data from a unique *multilateral sample*—that is, sojourners from multiple origins traveling to multiple destinations; this provided the opportunity to investigate the contextual effects of the countries of origin and destination simultaneously. Moreover, by employing a two-wave longitudinal design, the present study also examined whether the effect of cultural tightness on cultural adaptation is temporally variant or stable.

Finally, we were interested in the moderating role that personality may play in predicting sojourner success. There is a robust literature on personality and cultural adaptation, with findings pointing to adaptive aspects of extraversion, agreeableness, conscientiousness, openness, and honesty-humility from the five-factor and HEXACO models of personality, as well as the positive impact of more narrowly defined traits such as cultural empathy (Demes & Geeraert, 2015; Van Oudenhoven & Van der Zee, 2002; Ward, Leong, & Low, 2004; Wilson et al., 2013); however, these studies have been limited to investigations of the main effects of personality on adaptive outcomes. In contrast, we adopted a person–situation interactionist perspective, advancing the notion that the tightness of the destination culture need not have a uniformly negative impact on sojourners’ cultural adaptation. We specifically hypothesized that the negative impact of cultural tightness on adaptation is dampened among individuals with personality traits that effectively enable them to learn new cultural norms and display culturally appropriate, normative behaviors. Accordingly, the ability to skillfully navigate strong norms is most likely to be found among people who have high levels of agreeableness (e.g., who are concerned with fitting in and cooperating with other people; Ashton & Lee, 2009), conscientiousness (e.g., who are self-efficacious and able to self-regulate; De Vries & Van Gelder, 2013), and honesty-humility (e.g., who feel little temptation to break rules; Ashton & Lee, 2009). Thus, we expected the relationship between tight norms in the destination culture and sojourners’ adaptation to be moderated by their personality attributes (Hypothesis 4). We also explored main effects and interactions with tightness of extraversion, emotionality, and openness but did not have specific predictions regarding these interactive effects.

## Method

### Design and participants

Data were analyzed from a longitudinal acculturation project (see Demes & Geeraert, 2015), in which 2,480 young adults (age: *M* = 17.0 years, *SD* = 1.4 years; 70% female) participating in an intercultural exchange program were surveyed over an 18-month period, from 2 months before to 6 months after the exchange. All participants were registered with AFS Intercultural Programs, a nonprofit, volunteer-based organization offering intercultural exchange programs. Typically, students are placed with a host family for the duration of their 8- to 10-month stay abroad, and during this time, they enroll at a local high school.

Sojourners were surveyed a total of nine times, but for the purpose of the current research, we focused on data collected during three waves (Time 1, Time 3, and Time 5) because our measures of interest were assessed at only these times. Waves occurred approximately 3 months before (Time 1), 2 weeks after (Time 3), and 5 months after (Time 5) arrival to the host country. At each wave, participants were invited by e-mail to visit the project website, log in, and complete an online survey. Previous studies of adaptation have rarely examined variables over time. Analyzing adaptation longitudinally provides us with additional data points per sojourner and the unique opportunity to examine whether the influence of tightness on adaptation is stable or changes over time.

Each sojourner in this study was traveling from 1 of 46 different home countries to 1 of 51 different host destinations. Scores of cultural tightness (Gelfand et al., 2011) were available for 23 of these countries.^1^ Many participants had at least one tightness score for either their home or the host country (*n* = 2,265), but only half had scores for both (*n* = 1,137). Finally, participants needed to have completed the initial predeparture survey (Time 1) and at least one of the critical surveys during the exchange (Times 3 and 5), resulting in a final sample of 889 sojourners. A breakdown of sample size per sending and hosting countries alongside the countries’ tightness score is provided in Table 1.

**Table 1.** Sample Size per Country Traveled From (Home) and Country Traveled to (Host), Ordered by Tightness Score

Country	Tightness	*n*	
		From	To
Malaysia	1.19	9	10
India	1.03	15	6
Norway	0.97	59	27
Turkey	0.92	31	9
Japan	0.86	36	42
China	0.82	38	35
Portugal	0.76	2	16
Austria	0.69	26	12
Mexico	0.68	17	11
Germany	0.65	155	113
Italy	0.65	214	54
Iceland	0.64	3	11
France	0.63	42	30
Hong Kong	0.63	20	3
Belgium	0.56	26	34
Spain	0.54	9	11
United States	0.54	69	341
Australia	0.42	3	18
New Zealand	0.40	25	30
The Netherlands	0.38	1	12
Venezuela	0.37	9	14
Brazil	0.34	70	37
Hungary	0.25	10	13

### Measures

Surveys were administered in 10 different languages (English, Chinese, French, German, Italian, Japanese, Portuguese, Spanish, Thai, and Turkish), covering those most commonly spoken among participants. More than 20 different concepts were recorded through the online surveys, but here we concentrate on only those measures relevant to the present research questions.

#### Cultural adaptation

Incorporating the core sociocultural and psychological dimensions of cross-cultural adaptation (Searle & Ward, 1990; Ward, 2001), we used two scales to assess sojourners’ experiences during the sojourn: once at the start (Time 3) and once halfway through the sojourn (Time 5). The 12-item Brief Sociocultural Adaptation Scale (Demes & Geeraert, 2014) was used to assess ease of behavioral adaptation to social and cultural elements of the host country (e.g., how to behave in public, style of clothes, what people think is funny) on a 7-point scale (both αs > .80). The 8-item Brief Psychological Adaptation Scale (Demes & Geeraert, 2014) was used to assess the emotional and psychological aspects specific to a cultural relocation (e.g., how often you have felt excited about being in the host country) on a 7-point scale (αs > .80). For the purpose of the present analyses, the items were collapsed into a single construct, following good reliability overall (both αs > .90).

#### Early return

Data regarding sojourners’ premature return home were acquired directly from the exchange program organizers. Early return was a dichotomous variable coded as 1 (student returned home early) or 0 (student did not return home early). Of the total sample, 4% of sojourners returned home early.

#### Personality

Sojourners’ personality was assessed before travel (Time 1) using the HEXACO inventory (Ashton & Lee, 2009). This six-factor structure measures honesty-humility (e.g., “I would never accept a bribe, even if it were very large”), emotionality (e.g., “I feel like crying when I see other people crying”), extraversion (e.g., “The first thing that I always do in a new place is to make friends”), agreeableness (e.g., “Most people tend to get angry more quickly than I do”), conscientiousness (e.g., “I often push myself very hard when trying to achieve a goal”), and openness to experience (e.g., “People have often told me that I have a good imagination”); all factors had good reliability (αs > .70, except agreeableness: α = .67). In the present article, we focus on honesty-humility, agreeableness, and conscientiousness because of their theoretical relevance.

#### Multicultural experience

Sojourners’ prior multicultural experience was assessed by the number of languages they spoke, previous sojourn experience (e.g., “Have you ever spent longer than 1 month in another country? Yes or no”), the family’s previous sojourn experience (e.g., “Has anyone in your family ever studied/worked abroad? Yes or no”), and the family’s previous hosting experience (e.g., “Has your family ever hosted an exchange student? Yes or no”).

#### Tightness scores

Tightness scores for both the home and host countries were not measured directly, but data were gathered from Gelfand et al. (2011). In their study, tightness, measured across 6,823 respondents from 33 countries, was shown to have high within-nation agreement and high between-nation variability. A home- and host-country tightness score was assigned to each participant; higher scores indicate higher levels of tightness.

## Results

The data were analyzed through a series of longitudinal multilevel models^2^ following the procedures proposed by Hox (2010) and using MLwiN software (Version 2.36; Rasbash, Browne, Healy, Cameron, & Charlton, 2016). Interactions were plotted using the method by Preacher, Curran, and Bauer (2006). Cultural adaptation was analyzed in a series of two-level models with time as the primary unit of analysis (Level 1; *n* = 1,595) nested within individuals at the highest level (Level 2; *n* = 889). First, a basic model was computed including time as a fixed variable (Time 3 vs. Time 5, coded 0 or 1, respectively). Grand-mean-centered explanatory variables were added in subsequent models.

For each model, the following control variables were added: sex, parents’ socioeconomic status, and measures of sojourners’ past multicultural experiences (Tadmor, Galinsky, & Maddux, 2012), including number of languages spoken, previous sojourn experience of the individual, previous sojourn experience of family members, and previous hosting experience of the family. Overall, male sojourners were better adapted than female sojourners (*p* = .002), but no other control variable reached significance (all *p*s > .30). More importantly, the addition of the control variables did not alter the results in any way, and so we do not report them further.

### Home- and host-country tightness

To analyze the association between home- and host-country tightness and adaptation, we first computed a null model with adaptation varying at Level 1 (deviance = 3,808.06, *df* = 3). The interclass correlation showed that 51% of the variance was at the individual level and the remaining 49% at the repeated measures level. The inclusion of time (Model 1; see Table 2) did not improve the model, χ^2^(1) = 1.13, *p* = .288, but time was retained as a control variable.

**Table 2.** Results of the Multilevel Analysis Models of Home- and Host-Country Tightness on Adaptation

Predictor	Model 1: deviance = 3,806.93 (df = 4), c2(1) = 1.13, p = .288			Model 2: deviance = 3,787.82 (df = 6), c2(2) = 19.11, p < .001			Model 3: deviance = 3,786.84 (df = 7), c2(1) = 0.98, p = .321		
	*b*	*SE*	*p*	*b*	*SE*	*p*	*b*	*SE*	*p*
Intercept	4.89	0.03	< .001	4.89	0.03	< .001	4.89	0.03	< .001
Time (linear)	0.03	0.03	.288	0.03	0.03	.315	0.03	0.03	.315
Tightness									
Home country				0.34	0.14	.019	0.33	0.14	.022
Host country				−0.54	0.16	.001	−0.52	0.16	.001
Home Country × Host Country							1.10	1.11	.321
Residual variance									
σ^2^_*e*_ (Level 1: time)	0.36	0.02	< .001	0.36	0.02	< .001	0.36	0.02	< .001
σ^2^_*u*_ (Level 2: individual)	0.37	0.03	< .001	0.36	0.03	< .001	0.36	0.03	< .001

Note: The intercept for each model reflects the expected outcome for Time 3 (coded 0) and the averages of all the other predictors.

The addition of tightness scores for sojourners’ home and host countries (Model 2; see Table 2) statistically improved the model, χ^2^(2) = 19.11, *p* < .001. Host-country tightness was negatively related to adaptation (*b* = −0.54, 95% confidence interval, or CI = [−0.84, −0.23]), demonstrating that sojourners who traveled to a tighter country were less adapted than those traveling to a looser country, confirming our first hypothesis. Independently, home-country tightness was positively related to adaptation (*b* = 0.34, 95% CI = [0.06, 0.62]), suggesting that sojourners originating from a tighter country had a higher level of overall adaptation while abroad, confirming the second hypothesis. Next, we examined the buffering effect of tightness of the home country on the negative relationship between destination-culture tightness and adaptation. To this end, the home-by-host-country-tightness interaction was added (Model 3; see Table 2) but failed to improve the model, χ^2^ < 1 (*b* = 1.10, 95% CI = [−1.07, 3.26]). We thus found no support for our third hypothesis.

To examine whether the relationship between tightness and adaptation was time variant, we ran subsequent analyses. Two-way interactions of time and tightness (Home-Country Tightness × Time and Host-Country Tightness × Time) were added to Model 2, but this did not significantly change the model (χ^2^ < 1). Interestingly, this suggests that the effect of tightness on adaptation was stable over time. The subsequent addition of the three-way interaction (Home-Country Tightness × Host-Country Tightness × Time) also did not significantly change the results, χ^2^ = 2.22, *p* = .136.

### Personality as moderator

To examine whether personality moderated the effect of home- and host-country tightness (Hypothesis 4), we computed a series of models for each personality factor. Following on from the previous analyses, we first added the personality trait in each model, followed by the interaction of the personality trait with the tightness scores for the home and host countries. The summary statistics for these analyses are provided in Table 3.

**Table 3.** Results of the Multilevel Analysis Models of the Interaction Effects of Personality by Home- and Host-Country Tightness on Adaptation

Predictor	Agreeableness						Conscientiousness						Honesty-humility					
	Model 4: deviance = 3,762.97 (df = 8), χ2(1) = 23.87, p < .001			Model 5: deviance = 3,757.30 (df = 10), χ2(2) = 5.67, p = .059			Model 4: deviance = 3,772.29 (df = 8), χ2(1) = 14.55, p < .001			Model 5: deviance = 3,770.45 (df = 10), χ2(2) = 1.83, *p* = .400			Model 4: deviance = 3,779.21 (df = 8), χ2(1) = 7.63, p = .006			Model 5: deviance = 3,773.20 (df = 10), χ2(2) = 6.00, *p* = .050		
	*b*	*SE*	*p*	*b*	*SE*	*p*	*b*	*SE*	*p*	*b*	*SE*	*p*	*b*	*SE*	*p*	*b*	*SE*	*p*
Intercept	4.89	0.03	< .001	4.89	0.03	< .001	4.89	0.03	< .001	4.89	0.03	< .001	4.89	0.03	< .001	4.89	0.03	< .001
Time (linear)	0.03	0.03	.291	0.03	0.03	.287	0.03	0.03	.321	0.03	0.03	.320	0.03	0.03	.316	0.03	0.03	.313
Tightness																		
Home country	0.29	0.14	.042	0.28	0.14	.046	0.35	0.14	.014	0.33	0.14	.022	0.35	0.14	.016	0.35	0.14	.015
Host country	−0.52	0.15	.001	−0.51	0.15	.001	−0.52	0.16	.001	−0.52	0.16	.001	−0.54	0.16	.001	−0.59	0.16	< .001
Home Country × Host Country	1.11	1.09	.307	1.17	1.09	.281	0.83	1.10	.449	0.82	1.10	.458	1.07	1.10	.332	1.32	1.10	.230
Personality trait																		
Trait	0.15	0.03	< .001	0.15	0.03	< .001	0.11	0.03	< .001	0.11	0.03	< .001	0.08	0.03	.006	0.09	0.03	.003
Trait × Home Country				0.19	0.17	.266				0.21	0.16	.188				0.11	0.18	.530
Trait × Host Country				0.45	0.20	.025				0.08	0.17	.628				0.44	0.18	.015
Residual variance																		
σ^2^_*e*_ (Level 1: time)	0.36	0.02	< .001	0.36	0.02	< .001	0.36	0.02	< .001	0.36	0.02	< .001	0.36	0.02	< .001	0.36	0.02	< .001
σ^2^_*u*_ (Level 2: individual)	0.34	0.03	< .001	0.34	0.03	< .001	0.35	0.03	< .001	0.35	0.03	< .001	0.35	0.03	< .001	0.35	0.03	< .001

Note: The intercept for each model reflects the expected outcome for Time 3 (coded 0) and the averages of all the other predictors.

#### Agreeableness

**Fig. 1.**
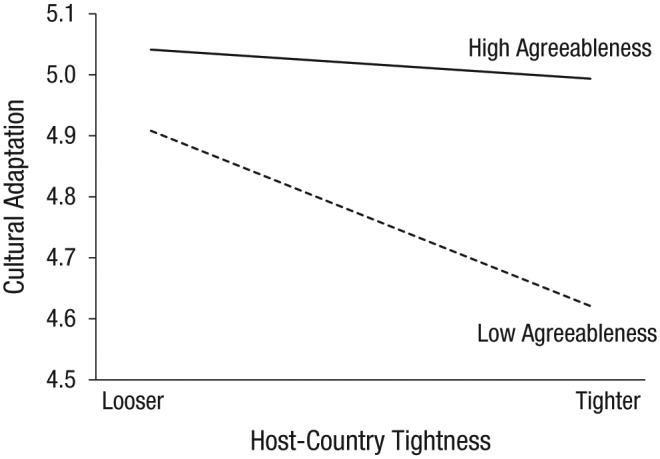
The relationship between host-country tightness and cultural adaptation as moderated by agreeableness.

The inclusion of agreeableness (agreeableness: Model 4) enhanced the basic model, χ^2^ = 23.87, *p* < .001, showing a positive relationship between agreeableness and adaptation (*b* = 0.15, 95% CI = [0.09, 0.21]). The addition of the trait-by-tightness interactions (agreeableness: Model 5) marginally improved the model, χ^2^(2) = 5.67, *p* = .059. At the level of individual predictors, the hypothesized interaction of agreeableness by destination-culture tightness emerged (*b* = 0.45, *p* = .025, 95% CI = [0.06, 0.84]), showing that agreeableness buffered the adverse effect of host tightness (see Fig. 1). Simple-slopes analyses were conducted to further examine the interaction. These analyses revealed that, compared with traveling to a looser host culture, traveling to a tighter destination was associated with lower adaptation for sojourners with low agreeableness (*p* < .001) but not for those with high agreeableness (*p* = .52).

#### Conscientiousness

Compared with the basic model, the model with the addition of conscientiousness was a significant improvement (conscientiousness: Model 4), χ^2^ = 14.55, *p* < .001; higher levels of conscientiousness were associated with higher levels of adaptation (*b* = 0.11, 95% CI = [0.05, 0.16]). However, the addition of the trait-by-tightness interaction (conscientiousness: Model 5) did not improve the model in any way, χ^2^(2) = 1.83, *p* = .40.

#### Honesty-humility

**Fig. 2.**
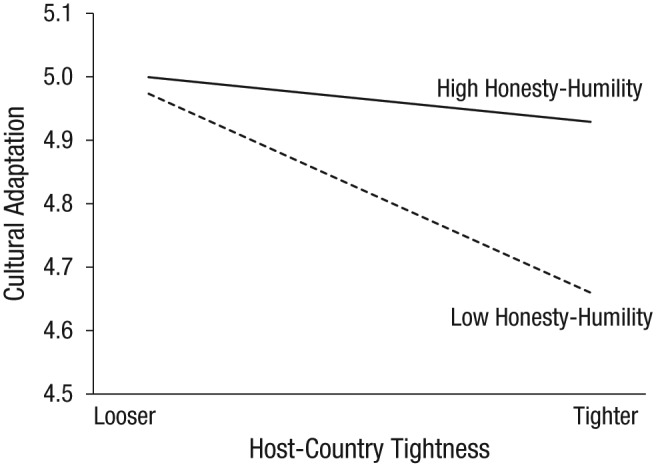
The relationship between host-country tightness and cultural adaptation as moderated by honesty-humility.

The inclusion of honesty-humility statistically improved the basic model (honesty-humility: Model 4), χ^2^ = 7.63, *p* = .006; higher levels of honesty-humility were associated with higher levels of adaptation overall (*b* = 0.08, 95% CI = [0.02, 0.14]). The addition of the trait-by-tightness interactions (honesty-humility: Model 5) slightly improved the model, χ^2^(2) = 6.00, *p* = .049. More importantly and as predicted, a trait-by-host-country-tightness interaction emerged (*b* = 0.44, *p* = .015, 95% CI = [0.09, 0.80]), showing that honesty-humility buffered the negative effect of the destination country’s tightness (see Fig. 2). Subsequent simple-slopes analyses showed that, compared with traveling to a destination with looser norms, traveling to a country with tighter norms was associated with lower adaptation for sojourners with low honesty-humility (*p* < .001) but not for those with high honesty-humility (*p* = .29).

#### Other personality factors

Although no a priori hypotheses were advanced for the interaction of tightness with emotionality, extraversion, and openness on adaptation, we examined these in an identical manner. For each personality factor, a multilevel model was constructed, building on Model 3. Emotionality was negatively associated with adaptation, χ^2^ = 43.27, *b* = −0.19, *p* < .001, 95% CI = [−0.24, −0.13], but the trait-by-tightness interactions were not significant (*p*s > .19). Similarly, although extraversion was positively associated with adaptation, χ^2^ = 59.27, *b* = 0.23, *p* < .001, 95% CI = [0.17, 0.29], the interactions were not significant (*p*s > .50). Finally, openness was positively associated with adaptation, χ^2^ = 4.94, *b* = 0.06, *p* = .026, 95% CI = [0.01, 0.12], but again, the trait-by-tightness interactions were not significant (*p*s > .20). In line with previous research, the analyses revealed a relationship between personality and adaptation: Higher adaptation was associated with higher levels of extraversion and openness and lower levels of emotionality. Importantly, none of the person–situation interactions were significant.

#### Host-country selection bias

Our analyses suggested that sojourners’ agreeableness and honesty-humility buffered the negative effect of traveling to a tight host country. An alternative explanation, however, is that, depending on their personality, people may choose certain countries. To assess this hypothesis concerning host-country selection bias, we examined the bivariate correlations between sojourners’ personality (before travel) and the tightness score of the host country. This revealed a modest negative correlation between extraversion and host-country tightness (*r* = −.09, *p* = .006), indicating that extraverted sojourners were more likely to go to a loose country. Crucially, however, no other effect emerged (*p*s > .05), indicating that there was no apparent relationship between host-country tightness and our key personality measures (agreeableness, conscientiousness, and honesty-humility).

### Early return

Finally, we examined the impact of home- and host-culture tightness on a behavioral outcome. Most of our participants stayed for the planned term of their exchange; however, 4% of sojourners, experiencing severe difficulties in adjustment, returned home early. This dichotomous behavioral measure (early return: yes vs. no) was analyzed by means of a binary logistic regression, in which early return was regressed on tightness scores of the home and host countries. The results showed that home-country tightness was negatively associated with early return, Wald χ^2^ = 8.28, *p* = .004, suggesting that sojourners coming from a tighter home country were significantly less likely to return home early compared with sojourners coming from a loose country. The effect of host-country tightness was not significant, Wald χ^2^ = 1.43, *p* = .232. The interaction of home- and host-country tightness was added in the final step, but the addition of the interaction did not improve the model, Wald χ^2^ < 1.

## Discussion

In this era of global interdependence, it is critical for theory and practice to identify the factors that facilitate versus hinder sojourner adjustment. This research introduced, for the first time, an interactionist approach to adjustment, wherein the home and host contexts along with personality affect adaptation outcomes. First, we illustrated that the strength of social norms is critical for understanding difficulties in adaptation.^3^ In this respect, quite clearly, the destination matters in that individuals sojourning to tighter cultures have poorer adaptation outcomes. Moreover, sojourners coming from tight cultures have better adaptation outcomes. They not only reported higher adjustment but also were less likely to return early compared with sojourners from loose cultures. Remarkably, the effect of both home-country tightness and host-country tightness proved stable over time.

Yet this research also illustrates the critical importance of an interactionist perspective; specifically, it is not inevitable that sojourners fare poorly when traversing to tight cultures. Rather, individuals who had high levels of agreeableness and honesty-humility were buffered from the negative impact of strong norms on adjustment. This suggests that the more one’s personality fits the demands of strong social norms, the better the adjustment.

More generally, this research illustrates that broad generalizations about individuals’ adaptation solely based on either individual differences or the nature of the cultural context are likely to be incomplete and that future research should focus on both in combination. It also opens up new and exciting avenues for research on culture, personality, and adjustment. For example, the current analysis could be extended to include other individual differences that have been shown to be related to the strength of social norms (Gelfand et al., 2011). In particular, we might expect that individuals with high levels of self-monitoring, prevention focus, impulse control, and need for structure (Gelfand et al., 2011) would adapt better in tighter cultures, in line with the findings of personality–cultural-norm-strength fit shown here. By contrast, individuals with a high tolerance of ambiguity, a promotion focus, and lower need for structure might adapt better in looser cultures. Future research should also examine whether the theory and research advanced here apply to adjustment within cultures that vary on tightness–looseness (e.g., Harrington & Gelfand, 2014). For example, it is possible that individuals moving from California to South Carolina, which have been identified as relatively loose and tight cultures, respectively, might follow patterns that are similar to those identified in this research. Likewise, we might find that individuals socialized in tight organizations (e.g., the military) will have an easier time adjusting to new cultures compared with individuals from loose organizations (e.g., start-ups).

To be sure, like all research, this study has strengths and limitations. Notable strengths are the multilateral sample, which allowed the simultaneous examination of normative context of both the destination country and country of origin, and the longitudinal design. A notable limitation is that the research is largely based on self-reports from one type of sojourning group (i.e., international students), although we did also analyze early return as a behavioral indicator of adjustment. Future research should be extended to expatriate and immigrant groups and incorporate a wider range of adjustment indicators (e.g., performance ratings from supervisors, peer evaluations, or cortisol levels for indication of stress) to complement self-reports. Future research should also investigate the mechanisms accounting for our results. For example, we might expect that people sojourning to tight cultures will experience greater isolation and less integration because of difficulties in fitting into the stronger norms characteristic of these cultures but that people with high levels of agreeableness and honesty-humility do not experience such effects. More broadly, our longitudinal survey research could be supplemented by lab-based experimental studies that examine the causal effects of tightness–looseness on psychological well-being and adaptive behaviors and how context interacts with personality characteristics.

This research also has important practical implications. With the rapid increase in international education and trade and the continuing movement toward globalization, the number of sojourners is likely to continue to increase. Poor adjustment is problematic and extremely costly for individuals, organizations, and society. Selection for international placements and assignments might be made with an eye toward choosing which individuals would best match the strength of the normative context. Furthermore, training programs could address issues pertaining to tight social norms and target individuals who are likely to experience more difficulties in tight cultural contexts. By ensuring a good fit between sojourner personality and the norms of the destination culture, we will be more likely to reap the benefits associated with the increased global mobility.

## Supplemental Material

GeeraertSupplementalMaterial – Supplemental material for A Tight Spot: How Personality Moderates the Impact of Social Norms on Sojourner Adaptation Click here for additional data file. Supplemental material, GeeraertSupplementalMaterial for A Tight Spot: How Personality Moderates the Impact of Social Norms on Sojourner Adaptation by Nicolas Geeraert, Ren Li, Colleen Ward, Michele Gelfand and Kali A. Demes in Psychological Science

## Notes

1. One of the regions in this study is Hong Kong, which is not a country but an autonomous territory of the People’s Republic of China. The word *country* is thus used loosely throughout the article to include countries and autonomous territories.

2. Longitudinal multilevel modeling is conceptually similar to latent growth modeling.

3. Further convergent evidence for the role of social norms comes from analyses on a different outcome measure—acculturative stress. These analyses are provided in the Supplemental Material available online.
