# Supplementary material for: A Tight Spot: How Personality Moderates the Impact of Social Norms on Sojourner Adaptation
Source: Psychol Sci. 2019 Jan 23;30(3):333–42. doi: 10.1177/0956797618815488 (PMC6419235; doi:10.1177/0956797618815488)
Supplement: GeeraertSupplementalMaterial – Supplemental material for A Tight Spot: How Personality Moderates the Impact of Social Norms on Sojourner Adaptation [file GeeraertSupplementalMaterial.pdf]

## Supplementary materials

The challenges associated with cultural transitions have long been associated with a stress response (Berry, 1997). In this light, acculturative stress signals the need to cope with the challenges of the new environment and prepares sojourners to respond to the new cultural reality. Complementing the analyses on cultural adaptation, the supplementary materials explore how the strength of social norms (tightness) of the destination country and the country of origin affect acculturative stress. Our hypotheses conceptually mirror those of cultural adaptation. Specifically, we hypothesize that sojourners who venture to a tighter culture will experience higher levels of stress than those that go to a looser culture (*Hypothesis 1*). We further predict that sojourners originating from a tighter culture will experience lower levels of stress than those that go to a tighter culture (*Hypothesis 2*). In terms of stress, we also hypothesize that home culture tightness will buffer the negative effect of host culture tightness (*Hypothesis 3).* Finally, from a person-situation interactionist perspective, we hypothesize that the negative impact of cultural tightness on stress will be lower among individuals with *high agreeableness*, *high conscientiousness*, and *high honesty-humility*. Thus, we expect an interaction effect between tight norms in the destination culture and personality attributes of sojourners to predict perceived stress (*Hypothesis 4*).

## Method

**Dataset.** The analysis in the supplementary materials consists of the same dataset as the main paper. Instead of cultural adaptation however, the main outcome variable in the analyses was perceived stress was examined as a proxy for acculturative stress. The other measures include tightness scores for the host and home countries and individual personality measures.

**Perceived stress.** At each timewave, participants responded to a brief version of the Perceived Stress Scale (Cohen, Kamarck & Mermelstein, 1983). Participants were asked 'In the last 2 weeks how often have you felt …' which was followed by 4 items such as 'you were unable to control the important things in your life'. The scale was administered in the language of the survey (one of 10 different languages) prior to travel (t1 and t2) and at approximately 2 weeks (t3), 2.5 months (t4), 5 months (t5) and 8.5 months (t6) after arrival to the host country. Participants responded using a 7 point scale (1 = never, 7 = always) and reliability at each wave was good (all $\alpha$'s > .70).

## Results

The data were analyzed through a series of longitudinal multilevel models. Stress was analyzed in a two-level model with time as the primary unit of analysis (level 1, $N$ = 3113) nested within individuals at the highest level (level 2, $N$ = 909)[1]. In each analysis, a basic model was computed including time (t3 to t6, coded 0 to 3). Grand-mean centered explanatory variables were added in subsequent models.

### Home and host country tightness

To analyze the association between home and host country tightness and adaptation, we first computed a null model with stress varying at level 1 (*Deviance* = 8481.23, *df* = 3). The interclass correlation showed that 49% of the variance was at the individual level and the remaining 51% at the repeated measures level. Next, time and baseline stress[2] were included (Model 1, see Table 1), which improved the model, $\chi^2$ =

[1] Sample size for the analyses on stress was slightly higher than the analyses on adaptation. Because stress was measured at each time point more data points were available resulting in lower attrition rates.

[2] The mean of t1 and t2 stress was taken as the baseline as it was deemed to be a more stable value than stress at either one of the two time points alone. However, conducting the analysis with a single time point baseline produced an identical pattern of results.

| | Model 1 | | | Model 2 | | | Model 3 | | |
|---|---|---|---|---|---|---|---|---|---|
| | *B* | *SE* | *p* | *B* | *SE* | *p* | *B* | *SE* | *p* |
| intercept | 2.91 | .02 | .000 | 2.91 | .02 | .000 | 2.91 | .02 | .000 |
| time (linear) | -.09 | .01 | .000 | -.09 | .01 | .000 | -.09 | .01 | .000 |
| stress baseline (t1) | .47 | .03 | .00 | .47 | .03 | .000 | .47 | .03 | .000 |
| tightness | | | | | | | | | |
| home country | | | | -.21 | .14 | .144 | -.20 | .14 | .152 |
| host country | | | | .52 | .15 | .00 | .51 | .15 | .001 |
| home x host | | | | | | | -.38 | 1.08 | .72 |
| residual variance | | | | | | | | | |
| $\sigma^2_e$ (L1: time) | .58 | .02 | .00 | .58 | .02 | .00 | .58 | .02 | .00 |
| $\sigma^2_u$ (L2: individual) | .38 | .03 | .00 | .37 | .03 | .00 | .37 | .03 | .00 |
| model statistics | | | | | | | | | |
| *Deviance* (*df*) | 8166.04 (5) | | | 8151.26 (7) | | | 8151.14 (8) | | |
| $\chi^2$ (*df*) | 315.19 (2) | | | 14.77 (2) | | | .13 (1) | | |
| *p* | .000 | | | .001 | | | .723 | | |

**Table 1.** Multilevel analyses of home and host country tightness on perceived stress

315.19, $p < .001$. More importantly, the addition of tightness scores for sojourners' home and host country (Model 2, Table 1) further improved the model, $\chi^2(2) = 14.77$, $p < .001$. Host country tightness was positively related to stress ($B = .52$, *95% CI* = [.22, .82]), implying that sojourners who travelled to a tighter country experienced higher levels of acculturative stress. As predicted, home country tightness was negatively related to stress, but this effect failed to reach significance ($B = -.21$, *95% CI* = [-.48, .07]). Next, the interaction of home by host country tightness was added (Model 3, Table 1), but this failed to improve the model, $\chi^2 < 1$ ($B = -.38$, *95% CI* = [-2.50, 1.73]).

**Personality as moderator**

To examine whether personality moderated the effect of home and host country

| | Agreeableness | | | | | | Conscientiousness | | | | | | Honesty-humility | | | | | |
|---|---|---|---|---|---|---|---|---|---|---|---|---|---|---|---|---|---|---|
| | Model 4 | | | Model 5 | | | Model 4 | | | Model 5 | | | Model 4 | | | Model 5 | | |
| | *B* | *SE* | *p* | *B* | *SE* | *p* | *B* | *SE* | *p* | *B* | *SE* | *p* | *B* | *SE* | *p* | *B* | *SE* | *p* |
| intercept | 2.91 | .02 | .000 | 2.91 | .02 | .000 | 2.91 | .02 | .000 | 2.91 | .02 | .000 | 2.91 | .02 | .000 | 2.92 | .02 | .000 |
| time (linear) | -.09 | .01 | .000 | -.09 | .01 | .000 | -.09 | .01 | .000 | -.09 | .01 | .000 | -.09 | .01 | .000 | -.09 | .01 | .000 |
| stress baseline (t1) | .46 | .03 | .000 | .46 | .03 | .000 | .45 | .03 | .000 | .45 | .03 | .000 | .46 | .03 | .000 | .46 | .03 | .000 |
| tightness | | | | | | | | | | | | | | | | | | |
| home country | -.18 | .14 | .20 | -.18 | .14 | .20 | -.22 | .14 | .12 | -.20 | .14 | .17 | -.21 | .14 | .144 | -.17 | .14 | .22 |
| host country | .51 | .15 | .001 | .51 | .15 | .001 | .51 | .15 | .001 | .51 | .15 | .001 | .52 | .15 | .001 | .56 | .15 | .000 |
| home x host | -.40 | 1.07 | .71 | -.39 | 1.08 | .72 | -.22 | 1.08 | .84 | -.21 | 1.08 | .85 | -.38 | 1.08 | .726 | -.60 | 1.08 | .58 |
| personality trait | | | | | | | | | | | | | | | | | | |
| trait | -.08 | .03 | .01 | -.08 | .03 | .006 | -.08 | .03 | .01 | -.08 | .03 | .004 | -.03 | .03 | .33 | -.03 | .03 | .34 |
| trait x home | | | | .01 | .17 | .93 | | | | -.18 | .16 | .26 | | | | .27 | .18 | .13 |
| trait x host | | | | -.04 | .20 | .82 | | | | -.10 | .17 | .54 | | | | -.39 | .18 | .03 |
| residual variance | | | | | | | | | | | | | | | | | | |
| $\sigma^2_e$ (L1: time) | .58 | .02 | .00 | .58 | .02 | .00 | .58 | .02 | .00 | .58 | .02 | .00 | .58 | .02 | .00 | .58 | .02 | .00 |
| $\sigma^2_u$ (L2: individual) | .37 | .03 | .00 | .37 | .03 | .00 | .37 | .03 | .00 | .37 | .03 | .00 | .37 | .03 | .00 | .36 | .03 | .00 |
| model statistics | | | | | | | | | | | | | | | | | | |
| *Deviance* (*df*) | 8143.48 (9) | | | 8143.41 (11) | | | 8143.41 (9) | | | 8141.94 (11) | | | 8150.20 (9) | | | 8142.31 (11) | | |
| $\chi^2$ (*df*) | 7.66 (1) | | | .06 (2) | | | 7.73 (1) | | | 1.47 (2) | | | .93 (1) | | | 7.89 (2) | | |
| *p* | .006 | | | .969 | | | .005 | | | .479 | | | .334 | | | .019 | | |

**Table 2.** Multilevel analyses of personality by home and host country tightness interaction on perceived stress

tightness we computed a series of models for each personality factor. Following on from the previous analyses, in each model the personality trait was added first, followed by the interaction of the personality trait with the tightness scores for the home and host country. The summary statistics for all analyses are provided in Table 2.

**Agreeableness.** The inclusion of agreeableness (Agreeableness - Model 4) enhanced the basic model, $\chi^2 = 7.66$, $p < .01$, showing a negative relationship between agreeableness and stress ($B = -.08$, *95% CI* = [-.14, -.02]). However, the addition of the trait by tightness interactions (Agreeableness - Model 5) did not improve the model, $\chi^2 < 1$, and the predicted interaction of agreeableness by destination culture tightness did not emerge ($B = -.04$, *95% CI* = [-.43, .34]).

**Conscientiousness.** Compared to the basic model, the addition of conscientiousness was a significant improvement (Conscientiousness - Model 4), $\chi^2 = 7.73$, $p < .01$, such that higher levels of conscientiousness were associated with lower levels of stress ($B = -.08$, *95% CI* = [-.13, -.02]). However, the trait by tightness interactions (Conscientiousness - Model 5) did not improve the model, $\chi^2(2) = 1.47$, $p = .48$, nor did the interaction of conscientiousness by destination culture emerge ($B = -.10$, *95% CI* = [-.43, .23]).

**Honesty-humility.** The inclusion of honesty-humility, did not improve the basic model (Honesty-humility - Model 4), $\chi^2 < 1$ ($B = -.03$, *95% CI* = [-.09, .03]). More interestingly however, the addition of the trait by tightness interactions (Honesty-humility - Model 5) did improve the model, $\chi^2(2) = 7.89$, $p < .02$. As predicted, a trait by host country tightness interaction emerged ($B = -.39$, *95% CI* = [-.74, -.04]), showing honesty-humility to buffer the negative effect of the destination country (see Figure 1). Compared to going to a looser culture, sojourners going to a tighter culture reported higher levels of stress for sojourners

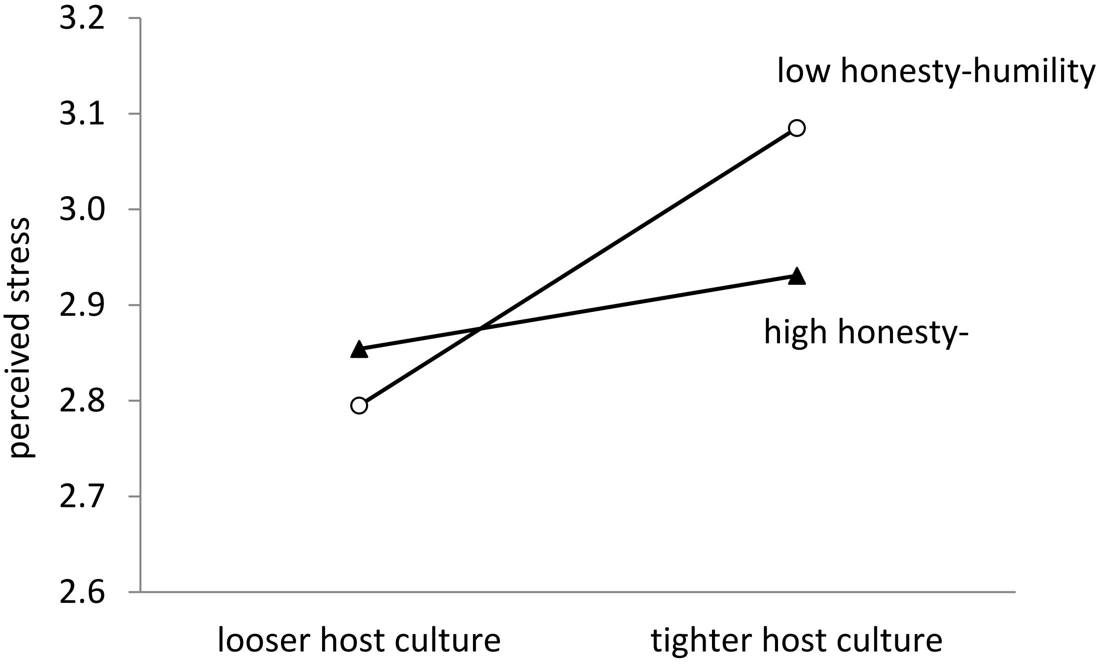


**Figure 1.** The relationship between host country tightness and perceived stress moderated by honesty-humility

who scored scoring low on honesty-humility ($p < .001$), but not for those that score high on the trait ($p = .24$).

**Discussion**

Looking at acculturative stress as an alternative outcome variable, we set out to conceptually replicate the analyses on cultural adaptation. The majority of results on stress were in line with those of cultural adaptation. First, host country tightness was predictive of higher levels of stress. Second, home country tightness was negatively related to stress, but not significantly so. There was no indication of a host by home interaction on stress, which matches the analyses on cultural adaptation. In terms of the personality-situation interaction, we did replicate the interaction of honesty-humility by host culture tightness on stress. More specifically, the relationship between host country tightness and acculturative

stress was moderated by honesty-humility, thereby conceptually replicating the earlier analyses. As a set, the analyses on acculturative stress provide further congruent evidence for the role of social norms in cultural transitions.

## References


Berry, J. W. (1997). Immigration, acculturation, and adaptation. *Applied Psychology, 46,* 5–68.

Cohen, S., Kamarck, T., & Mermelstein, R. (1983). A global measure of perceived stress. *Journal of Health and Social Behavior, 24(4)*, 385–396.
